# Graphene oxide integrated silicon photonics for detection of vapour phase volatile organic compounds

**DOI:** 10.1038/s41598-020-66389-9

**Published:** 2020-06-12

**Authors:** H. C. Leo Tsui, Osamah Alsalman, Boyang Mao, Abdullah Alodhayb, Hamad Albrithen, Andrew P. Knights, Matthew P. Halsall, Iain F. Crowe

**Affiliations:** 1 Photon Science Institute and Department of Electrical and Electronic Engineering, The University of Manchester, Manchester, United Kingdom M13 9PL; 2 National Graphene Institute and Department of Physics and Astronomy, The University of Manchester, Manchester, United Kingdom M13 9PL; 3 Aramco Laboratory for Applied Sensing Research, King Abdullah Institute for Nanotechnology, King Saud University, Riyadh, 11451 Saudi Arabia; 4 Department of Engineering Physics and Centre for Emerging Device Technology, McMaster University, Hamilton, L8S 4L7 Ontario Canada

**Keywords:** Imaging and sensing, Optical spectroscopy

## Abstract

The optical response of a graphene oxide integrated silicon micro-ring resonator (GOMRR) to a range of vapour phase Volatile Organic Compounds (VOCs) is reported. The response of the GOMRR to all but one (hexane) of the VOCs tested is significantly higher than that of the uncoated (control) silicon MRR, for the same vapour flow rate. An iterative Finite Difference Eigenmode (FDE) simulation reveals that the sensitivity of the GO integrated device (in terms of RIU/nm) is enhanced by a factor of ~2, which is coupled with a lower limit of detection. Critically, the simulations reveal that the strength of the optical response is determined by molecular specific changes in the local refractive index probed by the evanescent field of the guided optical mode in the device. Analytical modelling of the experimental data, based on *Hill-Langmuir* adsorption characteristics, suggests that these changes in the local refractive index are determined by the degree of molecular cooperativity, which is enhanced for molecules with a polarity that is high, relative to their kinetic diameter. We believe this reflects a molecular dependent capillary condensation within the graphene oxide interlayers, which, when combined with highly sensitive optical detection, provides a potential route for discriminating between different vapour phase VOCs.

## Introduction

VOCs are hazardous materials mainly produced from industrial processes, including petroleum and oil refineries, paints and plastic production. VOCs generally have high vapour pressure and low boiling point, and they are emitted in the form of vapours that can cause serious problems to human health and the environment. Aromatic compounds such as benzene, toluene, xylene (BTX) are toxic and carcinogenic while alcohols and ketones can cause nervous system depression at high concentration^1,2^. Moreover, halogenated VOCs contribute to the greenhouse effect and ozone depletion^3^. Therefore, it is essential to be able to provide effective sensing methods capable of detecting, and importantly distinguishing between, these harmful vapours for both industrial processes and environmental monitoring.

Silicon photonics micro-ring resonators (MRRs) have emerged as one of the more promising sensing platforms in recent years because of their capacity for high sensitivity, small footprint and mass-scalable potential. The sensing modality of these optical waveguide based devices relies on the interaction between the evanescent field of the cavity guided mode and the surrounding medium. For a sufficiently strong analyte-surface interaction, a change in the effective refractive index, n_eff_ for the cavity mode is induced and this is reflected as a resonance wavelength shift in the optical transmission spectrum of the MRR. So-called *slotted* MRRs, consisting of two concentric ring waveguides separated by a narrow slot, have been employed in recent years for biochemical sensing after it was shown that the guided mode is confined within the low index slot, which provides for a stronger interaction between the guided mode field and the near-surface target (vapour or gas) molecules, leading to a higher detection sensitivity^4–7^. In addition, specifically for chemical vapour and gas sensing, thin polymer or inorganic coatings have also been employed as a transduction layer to capture and concentrate molecules close to the waveguide surface^8^, again leading to stronger light-matter interaction, with some reportedly capable of sub-ppm level detection^9–12^. The transduction layer can be a porous material or a material that preferentially reacts with, or binds to, specific target molecules.

Engineered carbon materials, for example activated carbon and carbon nanotubes have been popular material choices for VOC detection because of their high adsorption capacity and thermal stability^2^. Surface area is one of the major factors affecting the adsorption performance, and the high surface-to-volume ratio makes graphene and its derivatives, e.g. graphene oxide (GO) ideal candidates for such applications^13–17^. Both graphene and GO demonstrate exceptional molecular permeation properties meaning they can be used as a molecular sieve, which has led to various applications^18–22^. Although single layer graphene is nonporous, because of its hydrophobic nature, the stacking of graphene nano-sheets can form mesopores, which have demonstrated exceptional water vapour adsorption as a result of the capillary condensation^23,24^. Similarly, experiments and molecular dynamics simulations have confirmed multilayer water formation in GO laminates by capillary condensation in the interlayer space^25^. From the results presented in this work, we suggest that VOCs undergo a similar condensation mechanism in GO. This study explores the sensing potential of a GO integrated silicon MRR, with focus on VOC vapour detection.

### Experimental and simulation details

**Figure 1:**
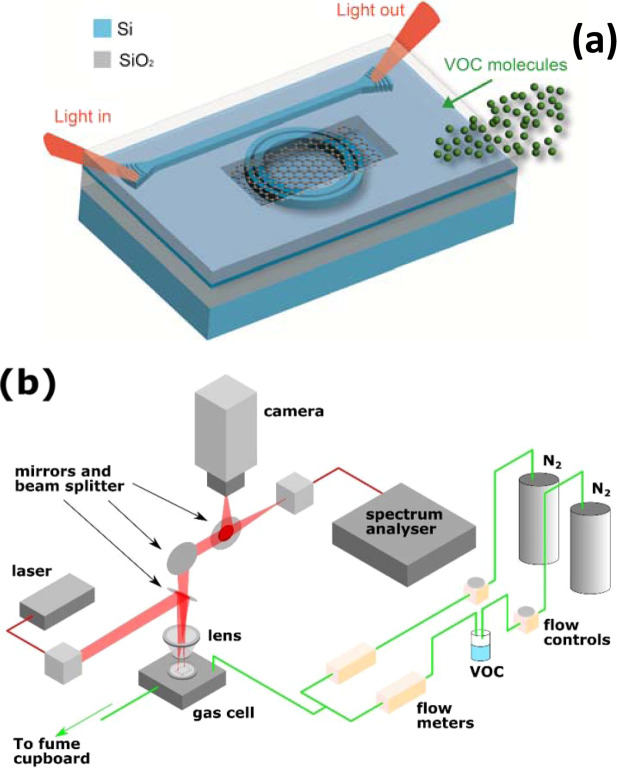
Schematic of (**a**) GO integrated micro-ring resonator exposed to VOC molecules and (**b**) Optical characterisation and vapour delivery setup.

The MRR employed in this study is an oxide capped, slotted ring with an outer waveguide width of 250 nm, slot width of 200 nm and an inner waveguide width of 290 nm. The MRR has a radius of 25.35 µm, measured from the ring centre to the extent of the outer waveguide. Light is coupled to the MRR from a straight bus waveguide with a 320 nm width and a slab thickness of 90 nm. The bus-to-outer ring coupling distance is 200 nm. A 64 µm × 35 µm window was opened in the oxide cap above the MRR, post fabrication, by selective hydrofluoric (HF) acid etching. Diffraction gratings (with 34 periods, each of length, Λ = 612 nm) are used to efficiently couple light into and out of the bus waveguide from near normal incidence. The device was fabricated in a commercial silicon foundry, using electron beam lithography, from a standard 220 nm silicon-on-insulator (SOI) starting wafer with a 2 µm buried oxide (BOX). Figure 1(a) illustrates the device structure.

GO sheets were exfoliated from graphite oxide using a procedure adapted from the published Hummers method^26^. Precise details of the GO preparation and solvent dispersion employed in this work have been described previously, elsewhere^27^. A total of 0.6 µl of 0.05 mg/ml GO solution was deposited on the MRR using a micropipette. To confirm the successful deposition of the GO on top of the MRR structure, a spatial Raman map of the GO related G and D peaks was taken from the window area (64 µm x 35 µm) in the oxide above the silicon MRR. A spatial resolution of 1 µm over the entire window area was obtained for the Raman map, using a Renishaw *inVia* spectrometer with a 633 nm laser source and a 50 ×(0.75 NA) microscope objective lens. The uncoated (control) silicon MRR and graphene oxide integrated silicon MRR are referred to as MRR and GOMRR respectively throughout the following discussion.

Optical waveguide transmission measurements were performed using a near-IR (1550 nm) single mode fibre-coupled broadband semiconductor laser diode, SLD (Thorlabs S5FC1005S) and an optical spectrum analyser (Thorlabs OSA203B). The light from the SLD was directed onto the input grating coupler of the bus waveguide through a collimator, mirror and a wide field of view, long working distance microscope objective lens and the transmitted signal was collected through the same lens and directed to the OSA via collection optics and single mode fibre. The spectrum was collected using the Thorlabs OSA software (version 2.4) with a high resolution and low-medium sensitivity setting, providing the accuracy and speed compromise necessary to make dynamic measurements of the MRR cavity wavelength shift with sufficient spectral resolution. The resonance wavelengths of the MRR/GOMRR were determined by Lorentzian fitting of the notches in the transmission spectra. The VOC vapours were generated using a custom built delivery system, which is comprised of two gas flows; a dilution flow and a vapour flow, which are combined before delivery to the gas cell for optical sensing measurements. The dilution flow was derived directly from a cylinder providing 99.998% pure N_2_ and its flow rate was maintained and monitored using a digital flow meter (SMC PFM750S-C8-A-W). The vapour flow was generated by evaporating VOC solvent in a vial, using a purpose built bubbler arrangement. The physical properties of the VOCs tested in this study are listed in Table 1. In all cases, a 15 ml vial was filled with 10 ml of solvent. The cap of the vial has two holes for connecting tubes; one inlet for the cylinder derived pure N_2_ (noting that N_2_ for dilution and vapour carrier were from two separate cylinders) and one outlet for vapour flow. The outlet vapour flow rate from the bubbler was varied by adjusting the inlet N_2_ flow rate and monitored using a digital flow meter (SMC PFM710S-C4-B-W).

**Table 1:** Physical properties of VOCs tested in this study.

VOC	Vapour pressure (Torr at 20°C)^59^	Molecular weight (g/mol)^60^	Kinetic diameter (Å)^61–64^	Relative Polarity^65^	Refractive index (RI) at 1.55 µm^66^
m-xylene	6.16	106.16	6.8	0.074	1.477
ethylbenzene (EB)	7.08	106.17	5.8	0.074	1.476
Water	17.545	18.02	2.6	1	1.316
Toluene	21.83	92.14	5.3	0.099	1.476
Ethanol	44.60	46.07	4.5	0.654	1.352
Benzene	75.20	78.11	5.3	0.111	1.479
Hexane	121.39	86.18	4.3	0.009	1.369
tetrahydrofuran (THF)	129.64	72.11	6.3	0.207	1.397
Acetone	185.45	58.08	4.6	0.335	1.348

For the vapour sensing measurement, the MRR device was placed in a custom built gas cell, with an inlet connected to the combined dilution and vapour flow tubes and the outlet was directed to a fume cupboard. The optical spectrum from the OSA was collected every 3 seconds with the setup described above. The dilution flow rate was kept at 1 l/min throughout the measurement, while the vapour flow rate was varied between 0 and 0.5 l/min in order to generate different vapour concentrations. Figure 1(b) shows the schematic diagram of the experimental setup.

**Figure 2:**
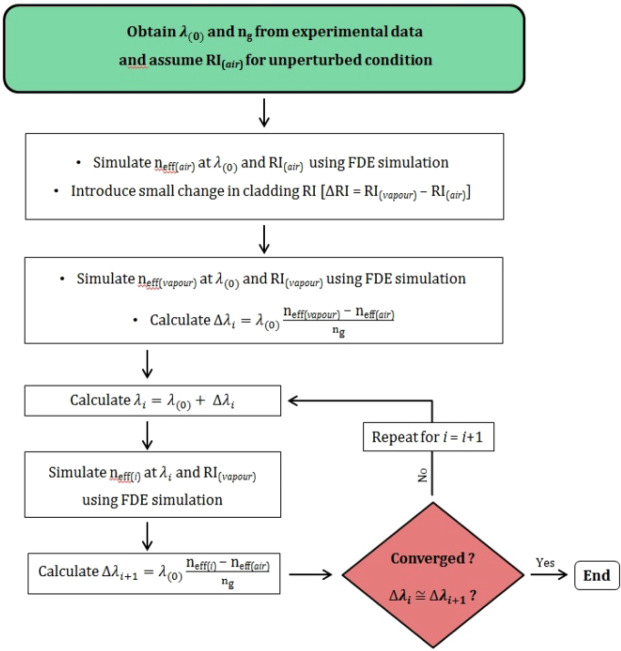
Flow chart of the iterative FDE simulation process for determination of the MRR device sensitivity.

In order to determine the device sensitivity, we calculated the resonance wavelength shifts for a particular change of refractive index in the top cladding layer (either air for the uncoated MRR or GO for the GOMRR) above the optical waveguide, using Eq. 1 and the iterative method described in^28^. The guided mode effective index was calculated using the Finite Difference Eigenmode (FDE) solver in Lumerical. In the FDE simulation, the refractive index of Si and SiO_2_ were set as 3.48 and 1.44 respectively and the waveguide dimensions were set as per the fabricated devices. The initial top cladding refractive index was set to 1 for the uncoated MRR and 2^29^ for the GOMRR, while the unperturbed resonance wavelength ${\lambda }_{(0)}$ and group indices ${{\rm{n}}}_{{\rm{g}}}\,({\lambda }_{(0)})$ were obtained from experimental data (details given in the results section). The iterative FDE simulation was performed until the resonance wavelength shift converged within $5\times {10}^{-3}\,$nm. The process flow of the iterative simulation is illustrated in Fig. 2, with i ∈ {0, 1, 2…} representing the number of iterations.

1 $$\Delta {\lambda }_{res}=\frac{\Delta {n}_{{\rm{eff}}}(\Delta {\rm{RI}},\Delta {\lambda }_{res})}{{{\rm{n}}}_{{\rm{g}}}\,({\lambda }_{(0)})}{\lambda }_{(0)}$$

## Results and Discussion

**Figure 3:**
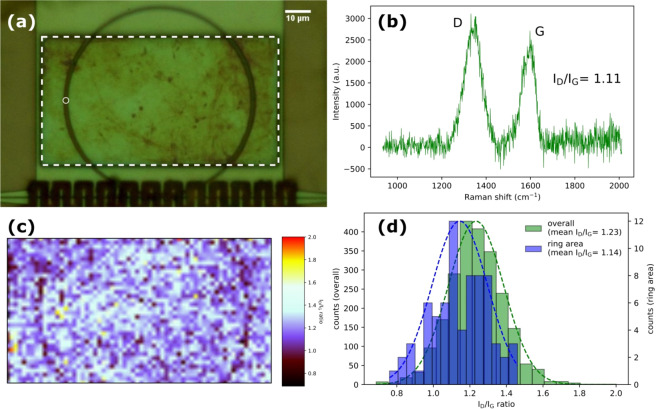
(**a**) Optical micrograph of GOMRR, showing the window area highlighted by the white dashed box; (**b**) A Raman spectrum obtained from the ring area as indicated by the white circle in (**a**); (**c**) Raman map of the I_D_/I_G_ ratio for the highlighted window area of the MRR; (**d**) Histograms and Gaussian fits (dotted lines) of the Raman I_D_/I_G_ ratio determined from fits to the Raman spectra for each mapped spatial positon.

An optical micrograph of the MRR is shown in Fig. 3(a) with GO deposited on the window area. Although GO covers the entire window area, only the GO covering the ring interacts strongly with the evanescent field of the guided optical mode and hence affects the sensing performance. An example Raman scattering spectrum obtained from the ring is shown in Fig. 3(b). GO is commonly characterised by the relative intensity of the D band to that of the G band in its Raman scattering spectrum. Specifically, the D to G band intensity ratio (I_D_/I_G_) is typically used to determine the oxidation level of GO^30^. The Raman map of I_D_/I_G_, Fig. 3(c) reveals a distribution of GO in the window area with a mean value of 1.23, which is within the reported range, 0.67 to 1.4^31^. However, a trace of darker colour from the map reveals the shape of the underlying ring, indicating a slightly lower I_D_/I_G_ ratio close to the ring. The statistical analysis, Fig. 3(d) confirms this with a mean I_D_/I_G_ ratio of 1.14 for the GO over the ring. Wroblewska *et al*. previously showed that the I_D_/I_G_ ratio increases as the GO undergoes thermal reduction^31^. However, the I_D_/I_G_ ratio is also known to be electrically tuneable^32^, suggesting that it is influenced by localised (trapped) charges, e.g. at the GO/silicon waveguide interface, which could explain the difference we observe here for the GO close to the MRR structure^33^.

**Figure 4:**
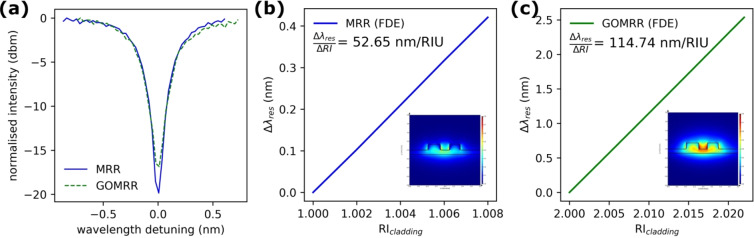
(**a**) Comparative optical transmission spectra of the MRR (blue solid line) and GOMRR (green dashed line); simulated resonance wavelength shifts for (**b**) MRR and (**c**) GOMRR. The insets show the TE mode electric field distributions (equal colour scales) associated with the guided optical mode in the slot waveguide based MRR device for the unperturbed condition.

A typical resonance from the transmission spectra of the 25 µm MRR with and without GO is shown in Fig. 4(a). The spectra are well described using Eqs. (2) and (3), where *r* is the MRR radius, *n*_*eff*_ is the waveguide effective index, *λ* is the wavelength, *a* is the power attenuation coefficient, *t* is the self-coupling coefficient and *T* is the transmission intensity, respectively^34,35^.

2 $$T=\frac{{a}^{2}-2at\,\cos \,\phi +{t}^{2}}{1-2at\,\cos \,\phi +{(at)}^{2}}.$$

3 $$\phi =\frac{4{\pi }^{2}r{n}_{eff}}{\lambda }.$$

The quality factor (λ/Δλ) of the MRR cavity resonance decreases from ~9000 to ~7500 after GO deposition. This relatively small change suggests that the absorption of light by the GO is not as strong as for graphene^33,36^, which is a key aspect for a functional layer for MRR based gas or vapour sensing, where a high quality factor is preferred, for a given detection resolution, in order to be able to measure relatively small shifts in the resonant wavelength.

In order to determine the theoretical resonance shift of the device for sensitivity analysis, the unperturbed resonance wavelength λ_0_ and group index n_g_ are required. These parameters can be extracted from the device transmission spectra obtained before introducing VOC test vapours. The unperturbed resonance wavelengths were determined precisely, by fitting the device transmission spectra, to be 1552.21 nm for the MRR and 1554.33 nm for the GOMRR. The corresponding group indices were determined from Eq. 4^34^, to be 3.404 for the MRR and 3.388 for the GOMRR.

4 $${{\rm{n}}}_{{\rm{g}}}({\lambda }_{0})=\frac{{\lambda }_{0}^{2}}{FSR\times L}.$$

Where FSR is the free spectral range and L (=2πr) is the physical length of the ring resonator. By incrementally changing the refractive index of the top cladding in the simulation, the corresponding resonance wavelength shift is obtained and the device sensitivity can then be determined in the commonly quoted unit of wavelength shift/Refractive Index Unit (nm/RIU). As shown in Fig. 4(b,c), the sensitivity of the GOMRR is ~2 times higher than that of MRR. This can be attributed to the smaller refractive index contrast at the interface between the silicon waveguide (n = 3.48) and the top cladding (n = 2)^37,38^ in the GOMRR device, which leads to lower confinement of the guided mode and thus a relatively strong evanescent field in the top cladding region (insets in Fig. 4(b,c)).

**Figure 5:**
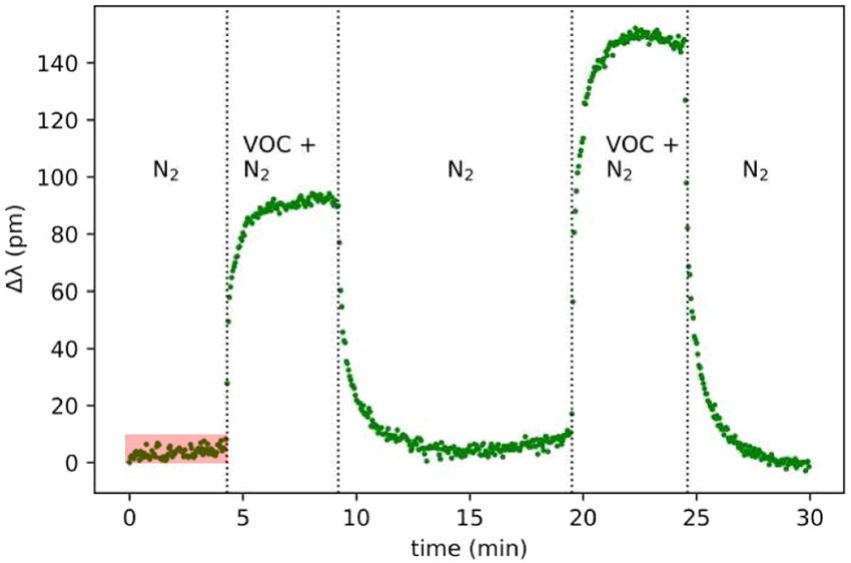
Time evolution of the resonance wavelength of the uncoated (control) MRR at different ethanol flow rates. The shaded box in the time range, 0 ≤ t ≤ 5 mins shows the approximate extent of the noise on the unperturbed resonance position (δλ ≤ 10 pm), which sets the limit of detection (LOD) for our sensing scheme.

Another key parameter in evaluating the ultimate capability of such a sensing device is the limit of detection (LOD). For a waveguide based device such as that employed in the present study, where we are specifically monitoring spectral changes in the MRR cavity resonance, due to changes in the top cladding (air or GO) refractive index, the LOD is proportional to the ratio of ‘distinguishable’ wavelength shift, δλ to the device sensitivity, S, i.e. LOD ∝ δλ/S^39^. Quantifying δλ is not trivial because, even in the absence of analyte driven changes in the MRR resonance (in our case carried in the vapour phase), the spectrum can still be perturbed by a variety of noise sources. In an attempt to account for these, so as to provide a conservative estimate for the absolute LOD in our devices, we use the general approach previously employed for similar MRR based devices^40^ in which δλ ≥ 3σ (i.e. the 99.7% range of uncertainty in determining the resonant peak position of the MRR). We have done this by monitoring the steady state (or equilibrium) MRR resonance position with time (shaded region in Fig. 5) and determine this to be ≤10 pm (for both the MRR and GOMRR). Taking S (=Δλ_eq_/ΔRI) to be the average values determined from Fig. 7 for the nine different VOCs tested (i.e. S_MRR_ = 52.57nmRIU^−1^ and S_GOMRR_ = 114.47nmRIU^−1^), yields an estimate for the LOD_MRR_ = 1/5257 = 1.9 × 10^−4^ RIU and LOD_GOMRR_ = 1/11447 = 8.7 × 10^−5^ RIU. These values are in generally good agreement with those previously reported for MRR based sensing devices^39,40^.

**Figure 6:**
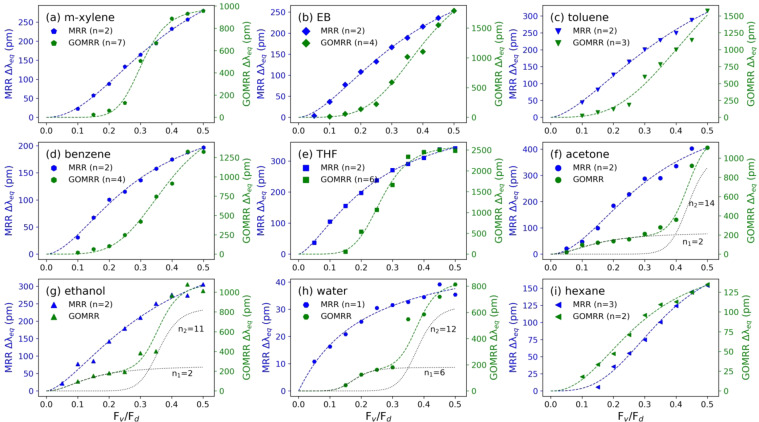
Equilibrium resonance shift (Δλ_eq_) as a function of vapour-dilution flow ratio (F_v_/F_d_) for various VOC vapours for MRR (blue) and GOMRR (green) with the Hill-Langmuir function (dashed lines).

**Figure 7:**
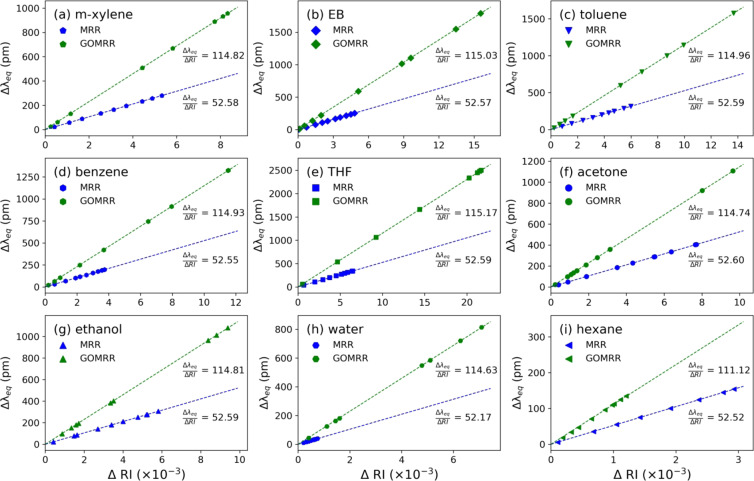
Equilibrium resonance shift (Δλ_eq_) as a function of change in refractive index for various VOC vapours for MRR (blue) and GOMRR (green).

In typical gas adsorption analysis, partial pressure and surface coverage are the commonly used terms, which are replaced by flow rate and resonance shift, respectively in the following discussion. The isotherm models can be adopted as the resonance wavelength shift reflects the change in refractive index of the cladding over the MRR, which can be viewed as the surface coverage, and thus the concentration of molecules on the bare (or GO coated) silicon surface. A typical change of MRR resonance wavelength in response to the presence of VOC is shown Fig. 5.

The equilibrium resonance shift (*Δλ*_*eq*_) with vapour concentration is illustrated in Fig. 6 and is well described by the *Hill-Langmuir* function, Eq. (5), where *x* is the vapour flow rate, *Δλ*_*max*_ is the maximum observed resonance shift, *k* represents the vapour flow rate at which half the maximum resonance shift is achieved and *n* is the *Hill* coefficient. This nonlinear function has previously been used to model the cooperative binding of oxygen to haemoglobin molecules and its application was also extended to other biochemical processes as well as surface adsorption of molecules^41–44^. In the context of biochemistry, the *Hill* coefficient is interpreted as being indicative of the degree of cooperative binding between ligand and macromolecules, while in this study *n* is considered as a parameter reflecting the level of cooperative adsorption^45–47^, with larger *n* values indicating increased cooperativity. That is to say that, for a receptor/substrate with multiple binding sites, the adsorption affinity of those binding sites for a particular molecule is increased once the binding of a single molecule is established.

5 $$\Delta {\lambda }_{eq}=\frac{{x}^{n}\Delta {\lambda }_{max}}{{k}^{n}+{x}^{n}}.$$

For the MRR, *Δλ*_*eq*_ increases in a gradual (hyperbolic) way with increasing vapour concentration (blue symbols in Fig. 6**)**, reflecting a low *n* value, indicative of low cooperativity, or perhaps even non-cooperative (independent) binding. This behaviour is similar to the low partial pressure regime in the *Langmuir* adsorption isotherm where molecular surface coverage is very low, possibly monolayer. A similar conclusion can be drawn from the perspective of the *Hill-Langmuir* model, where low *n* values (shown in the legend in each sub-plot) indicate low cooperativity and/or minimal multilayer adsorption. It is well known that the bare silicon surface has a ∼10 Å hydrophilic native oxide layer, where hydroxide is the main termination, which attracts organic compounds with low vapour pressure, light molecular weight or high polarity^48–50^. Therefore, the weak interaction between the silicon surface of the MRR and the VOC leads to a low surface coverage. Moreover, the presence of the continuous nitrogen flow maintains a cycle of adsorption/desorption of molecules from the silicon surface, which can limit multilayer adsorption, giving rise to a lower overall adsorption and cooperativity.

In contrast to the uncoated MRR, the integration of GO produces a sigmoidal line shape as *Δλ*_*eq*_ increases with increasing vapour flow rate (green symbols in Fig. 6). This relationship is consistent with the type V isotherm model, which indicates the presence of capillary condensation^51^. This is commonly found in porous media, where a vapour condenses at a pressure below its saturated vapour pressure^52–54^. The much higher *n* values obtained from the *Hill-Langmuir* fits in this case suggest that the cooperative adsorption is much more significant, i.e. higher probabilities of multi-site binding and multilayer formation. This supports the idea of capillary condensation, which can be separated into three stages: monolayer adsorption, multilayer adsorption and saturation^55^.

The Δ*λ*_*eq*_ produced by GOMRR at low concentration levels (represented by low flow ratio) is actually smaller than that of the uncoated MRR. This is attributed to the presence of the GO layer itself, which initially restricts the interaction between the near surface evanescent field of the guided optical mode and the target molecules. However, in the case of acetone, ethanol and water, because of their relatively small size the GO blocking is not as effective^56^, resulting in a similar behaviour to that of the MRR - with comparable *n* values, denoted as *n*_1_ in Fig. 6(f–h) - in this low concentration regime. Increasing the concentration of these VOCs helps establish the first monolayer, which is rapidly followed by multilayer adsorption^18,55^. The multilayer adsorption gives rise to the condensation in this confined pore structure, leading to a microscopic liquid environment surrounding the MRR. As the refractive index of liquid is higher than that of gas and vapour, this model of adsorption should lead to a greater resonance shift, induced by the greater change in refractive index in the surrounding medium. The larger *n* values obtained for acetone, ethanol and water at high concentration levels (denoted as *n*_2_ in Fig. 6(f–h)) indicates significantly increased cooperativity, which could also be due to the relatively high polarity of these molecules, which is known to be a factor in the ability of a molecule to intercalate within the layered structure of GO^19,21^. The saturation at the highest concentration levels suggests that almost all of the GO pores/interlayer spaces are occupied and cannot incorporate any more molecules.

As shown in Fig. 6(i), unlike all of the other VOCs, no such enhancement is observed for the GOMRR over MRR throughout the range of hexane vapour flow rate, and the fits to both data-sets yield similar (relatively low) *Hill* coefficients. We attribute this to a lack of hydroxyl or carboxyl functional groups in hexane; there are various interaction mechanisms that can contribute to the molecular adsorption by GO, for instance H-bond, van der Waals, electrostatic and π-π interaction^13^, but in the case of hexane, the interaction between the acyclic saturated hydrocarbon and the hydrophilic GO is weak and thus no hexane multilayer can form on GO. On the

other hand, hydrophilic functional groups are also absent from benzene, but its ring structure can form π-π stacking with the basal plane of GO^57,58^, which could explain its stronger interaction compared with hexane.

With the device sensitivity determined from the iterative FDE simulation, the resonance wavelength shifts obtained from experiments can be converted to a change in the top cladding refractive index, Fig. 7. As the device is only sensitive to changes in the top cladding refractive index, i.e. the local environment surrounding the ring, the induced resonance shift only reflects the actual concentration of molecules within the sensing area with a sensitivity (nm/RIU) that is independent of the type of molecule introduced. As the GOMRR device was determined to have a sensitivity ~2x higher than the MRR, then for the same vapour flow rate one might expect the GOMRR resonance shifts to be simply twice that of the MRR, reflecting an equivalent change in the top cladding refractive index. However, as is shown in Fig. 7, the experimentally determined GOMRR resonance shifts are not simply a factor 2x larger than that for the MRR and this discrepancy must be accounted for. In Fig. 7, we show all of the shifts, determined as a function of change in top cladding refractive index, from the iterative FDE simulations. This reveals that, for the GOMRR, a much larger range of refractive index change is introduced for the same range of vapour flow rate. This very different range of refractive index reflects a difference in the vapour concentration actually ‘delivered’ to the device (i.e. detected by the optical cavity resonance shift) for the same vapour flow rate. It reveals that, for certain flow rates, the actual ‘delivered’ vapour concentration to the GOMRR is very different to that for the MRR. This explains the sigmodal behaviour observed for the GOMRR shift with flow rate, being the result of molecular ‘blocking’ (by the GO layers) at low vapour flow rates and ‘accumulation’, leading to capillary condensation (within the GO layers) at higher vapour flow rates.

**Figure 8:**
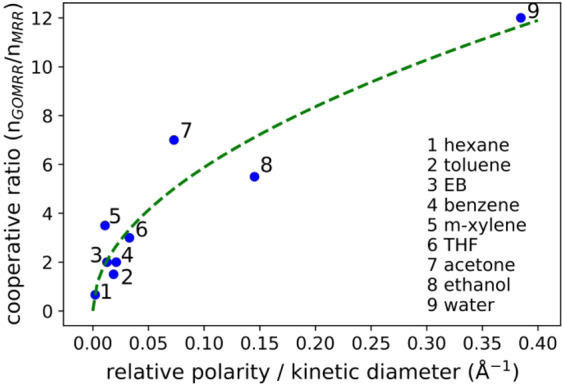
Cooperativity ratio (*n*_*GOMRR*_*/n*_*MRR*_) as a function of the relative polarity - kinetic diameter ratio for all VOCs tested, green dashed line is a guide to the eye.

Finally, if we consider the ratio of Hill coefficients for the GOMRR and MRR (control), in effect the cooperativity ratio (n_GOMRR_/n_MRR_) to be an indicator of molecular selectivity, we find that this is strongly correlated with molecular polarity (relative polarity) relative to kinetic diameter. Figure 8 indicates that it is the combined effect of polarity and size that leads to the efficient intercalation within GO layers and ultimately the capillary condensation in the confined pore structure of GO. As was previously demonstrated for GO, this essentially represents a method by which molecules may be discriminated, or selectively detected, for instance when they are present in a mixed vapour phase environment via what was termed ‘molecular sieving’^19,21^. Whilst further insight into the precise interaction between the different VOCs and the GO structure will undoubtedly be aided by e.g. Molecular Dynamics Simulations, the results we have presented here are consistent with the current understanding of this interaction, suggesting that GO as a functional layer, integrated with the silicon photonics platform, along with the underlying optical detection method we have applied, can provide an accurate and selective approach to VOC sensing.

## Conclusions

An enhanced VOC vapour sensing signal is obtained by integrating GO with a slotted silicon photonics waveguide based micro-ring resonator (MRR) device. The optical attenuation within the MRR is not significantly affected by the deposition of GO making it a potentially viable platform for functionalised optical sensing. The application of a straightforward *Hill-Langmuir* model is sufficient in describing the relationship between observed cavity resonance shifts and delivered vapour concentration for both the uncoated (control) MRR and the GOMRR. The relatively low, similarly valued *Hill* coefficients derived from this model for the uncoated MRR implies low-cooperativity or perhaps even non-cooperative (independent) molecular binding, i.e. where the adsorption and desorption kinetics are approximately equal. This suggests low, possibly monolayer, surface coverage with relatively low detection sensitivity and, critically, almost no way to discriminate between different vapour phase VOCs. However, we observe a marked increase in the optical response, i.e. the resonance shift with vapour flow rate, as well as a characteristic sigmoidal behaviour that is highly dependent on the VOC, when the same device is integrated with a GO functional layer. In this case, the data rather exhibits a type V isotherm-like relationship, which suggests that most of the VOC vapours undergo capillary condensation within the GO interlayer spaces. Iterative FDE simulations not only reveal a factor 2 improvement in the device sensitivity (nm/RIU) for the GOMRR, but also explain the observed sigmoidal behaviour in terms of molecular ‘blocking’ at low vapour flow rates and molecular ‘accumulation’ at higher vapour flow rates in the GO integrated device. This manifests in a much larger range of top cladding refractive index change for the GOMRR compared with the (control) MRR for the same vapour flow rate, which should be a critical consideration for devices functionalised in this way. For almost all of the VOCs we have tested, we observed an enhanced optical response. The exception to this, hexane, might be explained by the fact that it lacks either hydroxyl or carboxyl functional groups which, combined with the hydrophilic nature of GO, means that no hexane multilayer can form on GO. In general, the *Hill-Langmuir* analytical modelling of our data reveals an increase in molecular cooperativity (enhanced *Hill* coefficient) for the GO integrated device, further supporting the hypothesis of capillary condensation. We find that the ratio of *Hill* coefficients, n_GOMRR_/n_MRR_, is strongly correlated with a molecules polarity relative to its size (kinetic diameter), consistent with the current understanding that intercalation within the layered structure of GO is most efficient for highly polar molecules. Critically, this confirms GO’s property as a ‘molecular sieve’ and demonstrates that, in addition to signal enhancement, the integration of GO on silicon photonics MRRs can provide an effective route to selectivity in molecular detection.
